# Delayed Identification of Infants Who Are Deaf or Hard of Hearing — Minnesota, 2012–2016

**DOI:** 10.15585/mmwr.mm6911a6

**Published:** 2020-03-20

**Authors:** Abby C. Meyer, Melinda Marsolek, Nicole Brown, Kirsten Coverstone

**Affiliations:** ^1^Minnesota Department of Health; ^2^Children’s Minnesota Ear, Nose, and Throat, and Facial Plastic Surgery and Audiology, Minneapolis; ^3^University of Minnesota Department of Otolaryngology-Head and Neck Surgery, Minneapolis.

Few studies have examined factors associated with the timing of identification of hearing loss within a cohort of infants identified as deaf or hard of hearing (DHH) and what factors are associated with delayed identification. Minnesota Early Hearing Detection and Intervention (EHDI) personnel studied deidentified data from 729 infants with confirmed congenital hearing loss (i.e., hearing loss identification after not passing newborn hearing screening) born in Minnesota during 2012–2016. Differences in likelihood of delayed identification of congenital hearing loss (defined as not passing newborn hearing screening and age >3 months at the time of identification as DHH) based on multiple variables were analyzed. Overall, 222 (30.4%) infants identified as DHH had delayed identification. Multivariate regression showed that infants identified as DHH were significantly more likely to have delayed identification if they had 1) low birthweight, 2) public insurance, 3) a residence outside the metropolitan area, 4) a mother with a lower level of education, 5) a mother aged <25 years, or 6) a mother who was Hmong. Despite achievements of EHDI programs, disparities exist in timely identification of hearing loss. Using this information to develop public health initiatives that target certain populations could improve timely identification, reduce the risk for language delay, and enhance outcomes in children who are DHH.

The institution of EHDI programs has substantially reduced the average age of identification of infants who are DHH (*1*). Despite this, many infants do not meet the Joint Committee on Infant Hearing benchmark of identification of hearing loss by age 3 months (*2*). Although national EHDI data consistently show excellent screening rates, in 2016 only 75.9% of infants who did not pass screening had documentation of definitive diagnostic testing by age 3 months to identify whether a permanent hearing loss exists. Among those found to have a permanent hearing loss, only 67.3% were enrolled in Early Intervention services by the benchmark of age 6 months (*3*). Earlier enrollment in Early Intervention services among infants identified as DHH can improve language outcomes (*4*,*5*); however, a delay in identification of hearing loss might lead to delayed referral to Early Intervention and subsequently increase the risk for language delay in these children.

To determine characteristics associated with delayed diagnosis of hearing loss among Minnesota infants identified as DHH, data were collected by the Minnesota Department of Health (MDH) EHDI information system (EHDI-IS), which contains demographic, screening, diagnostic, and intervention data on children who have been identified as DHH. Data in EHDI-IS are obtained primarily from birth care providers and facilities, audiologists, public health nurses, and birth certificates via the Minnesota Office of Vital Records. The study population included 729 infants born in Minnesota during 2012–2016 who did not pass newborn hearing screening and were identified as DHH. Independent variables included location of residence and birth facility; maternal race/ethnicity, country of origin, age, and education level at the time of birth; primary language used in the home; birthweight; and infant’s health insurance status. If multiple maternal race categories were indicated on the birth certificate, the bridged race category derived by the Minnesota Office of Vital Records using the National Center for Health Statistics bridging methodology was used (*6*). Because Minnesota is home to some of the largest Somali and Hmong populations in the United States, Somali and Hmong were included as distinct populations. The outcome variable was delayed identification of hearing loss, defined as not passing newborn hearing screening and identification of hearing loss by 3 months of age. This study qualified as a public health program evaluation and therefore was considered exempt from Institutional Review Board review.

Prevalence ratios for delayed identification of hearing loss (age >3 months at identification compared with ≤3 months) were estimated for each independent variable using a modified Poisson regression model (*7*). Multicollinearity was assessed by testing for correlation coefficients >0.80 and variance inflation factors >2.5. Birth hospital location was highly correlated with residence location and therefore was not included in the multivariate model. Adjusted prevalence ratios were also estimated using a modified Poisson regression model (*7*). The final model used 686 records with complete data for all included variables. Statistical analyses were conducted in SAS software (version 9.4; SAS Institute).

Among 729 infants, 222 (30.4%) had delayed identification of hearing loss (Table). Bivariate analyses showed increased likelihood of delayed identification associated with residence location, birthweight, home language, maternal race/ethnicity, maternal country of origin, maternal age, maternal education level, and health insurance status.

**TABLE:** Characteristics associated with delayed identification of hearing loss (age >3 months) among infants identified as deaf or hard of hearing after not passing newborn hearing screening — Minnesota, 2012–2016

Characteristic (no. with available information*)	No.	No. (%) with delayed diagnosis	Prevalence ratio (PR) of delayed diagnosis	
			Unadjusted PR (95% CI)	Adjusted^†^ PR (95% CI)
**Total**	**729**	**222 (30)**	**—**	**—**
**Residence location at birth (698)**				
Metropolitan area^§^	411	112 (27)	Referent	Referent
Nonmetropolitan area	287	98 (34)	1.3 (1.0–1.6)^¶^	1.4 (1.0–1.8)^¶^
**Birth hospital location (699)**				
Metropolitan area^§^	463	130 (28)	Referent	**—**
Nonmetropolitan area	236	80 (34)	1.2 (1.0–1.5)	**—**
**Birthweight (698)**				
Normal (≥2,500g)	585	153 (26)	Referent	Referent
Moderately low (1,500g–2,499g)	85	34 (40)	1.5 (1.1–2.1)^¶^	1.4 (1.0–1.9)^¶^
Very low (<1,500g)	28	23 (82)	3.1 (2.5–3.9)^¶^	2.6 (2.0–3.3)^¶^
**Primary language used in the home (723)**				
English	609	181 (30)	Referent	Referent
Non–English	93	39 (42)	1.4 (1.1–1.8)^§^	1.1 (0.8–1.7)
American Sign Language	21	<5**	—**	0.2 (0.03–1.4)
**Maternal race/Ethnicity (695)**				
White	468	122 (26)	Referent	Referent
Hmong	69	32 (46)	1.8 (1.3–2.4)^¶^	1.6 (1.1–2.5)^¶^
Hispanic or Latina (of any race)	49	13 (27)	1.0 (0.6–1.7)	0.7 (0.4–1.2)
Black or African American (excluding Somali)	43	21 (49)	1.9 (1.3–2.6)^¶^	1.5 (1.0–2.3)
Asian (excluding Hmong)	34	10 (29)	1.1 (0.7–1.9)	1.2 (0.6–2.1)
Somali	21	5 (24)	0.9 (0.4–2.0)	0.7 (0.3–1.6)
American Indian	11	<5**	—**	1.1 (0.5–2.6)
**Mother born in the United States (698)**				
Yes	550	156 (28)	Referent	Referent
No	148	54 (36)	1.3 (1.0–1.7)^¶^	1.1 (0.7–1.6)
**Maternal age group (yrs) at birth (705)**				
<25	131	62 (47)	1.9 (1.5–2.4)^¶^	1.4 (1.1–1.8)^¶^
25–34	446	112 (25)	Referent	Referent
>34	128	40 (31)	1.2 (0.9–1.7)	1.3 (1.0–1.8)
**Maternal education level at birth (691)**				
College graduate or higher	246	40 (16)	Referent	Referent
Some college	233	83 (36)	2.2 (1.6–3.1)^¶^	1.6 (1.2–2.3)^¶^
High school graduate or less	212	85 (40)	2.5 (1.8–3.4)^¶^	1.7 (1.2–2.5)^¶^
**Public health insurance (infant) (729)**				
No	252	45 (18)	Referent	Referent
Yes	308	125 (41)	2.3 (1.7–3.1)^¶^	1.6 (1.1–2.2)^¶^
Unknown	169	52 (31)	1.7 (1.2–2.4)^¶^	1.4 (1.0–2.0)

**Abbreviation:** CI = confidence interval.

* For all variables except public health insurance, status was unknown if missing. Records with missing data in any of these variables were excluded from the multivariate analysis. Public health insurance status had a higher percentage unknown (23% versus 1%–5% for other variables) and thus a separate category of “unknown” was created to allow records with unknown public health insurance status to be included in the multivariate model.

^†^ The multivariate model was adjusted for residence location at birth, maternal race/ethnicity, mother born in the United States, maternal age, maternal education level, and public health insurance status.

^§^ Includes the seven counties (Anoka, Carver, Dakota, Hennepin, Ramsey, Scott, and Washington) of the Twin Cities region.

^¶^ Statistically significant (p<0.05).

** Number suppressed to protect data privacy.

After adjusting for other variables, the characteristic most strongly associated with delayed identification of hearing loss was birth weight, specifically very low birthweight (VLBW, <1,500g) or moderately low birth weight (MLBW, 1,500–2,499g). Overall, 82% of infants born with VLBW and identified as DHH received the diagnosis at age >3 months. Infants born with VLBW were more than twice as likely to have delayed identification, and infants born with MLBW also were significantly more likely to have delayed identification compared with infants born with normal birth weight (VLBW adjusted prevalence ratio [APR] = 2.6; 95% CI = 2.0–3.3); MLBW APR = 1.4; 1.0–1.8) (Table).

Maternal age and education also were significantly associated with delayed identification of hearing loss. Only 16% of infants born to a mother with a college degree were identified late, compared with 36% of infants born to a mother with some college and 40% born to a mother with a high school diploma or less. Infants born to mothers who did not have a college degree were more likely to be identified late (high school or less APR = 1.7; 1.2–2.5; some college APR = 1.6; 1.2–2.3). Approximately one half of infants born to a mother aged <25 years had delayed identification, and they were more likely to be identified late compared with infants whose mother was aged 25–34 years (APR = 1.4; 1.1–1.8) (Table). For the maternal race/ethnicity groups considered, infants whose mothers were Hmong were 60% more likely to have delayed identification compared with infants whose mothers were white (APR = 1.6; 1.1–2.5). Nearly one half of infants whose mothers were black had delayed identification. Although not significant at the p<0.05 level, the APR for this group was among the largest (APR = 1.5; 1.0–2.3). Infants whose residence was outside of the Twin Cities metropolitan area (APR = 1.4; 1.0–1.9) or who had public health insurance (APR = 1.6; 1.1–2.2) also were more likely to have delayed identification.

## Discussion

Socioeconomic factors are well documented determinants of health, and several socioeconomic indicators in this study were associated with delayed identification of infants who are born DHH. More work is needed to understand the barriers to audiologic follow-up for persons with lower socioeconomic status. Partnering with birth and primary care providers to improve messaging about the need for follow-up after newborn hearing screening and improvements in scheduling follow-up appointments for further testing at the time the infant does not pass the screening have both been identified as promising practices that might have a positive effect (*8*).

In this study, VLBW infants were at highest risk for delayed identification. These infants are at increased risk for multiple complications in the neonatal period, many of which are included in the Joint Committee on Infant Hearing’s list of risk factors for permanent congenital, delayed-onset, or progressive hearing loss in childhood (*2*). VLBW infants also might be medically fragile in the Neonatal Intensive Care Unit with acute issues that preclude conducting a diagnostic hearing test in a timely fashion. In addition, delayed identification was more likely for infants born to mothers of Hmong ethnicity. This finding has not been previously reported in the literature. In fact, a review of the literature revealed a paucity of hearing-related studies involving Hmong subjects (*9*). The reasons behind this association are unclear and further studies in this patient population are needed. Similar to previous findings (*10*), infants in this study living outside of the metropolitan area were more likely to have delayed identification compared with infants who live within the metropolitan area. Health care resources, particularly access to pediatric audiology services, might be limited in some nonmetropolitan regions. The development of tele-audiology programs to improve access has been well described and has been piloted in Minnesota with some success.* However, more work is needed to expand upon and further refine these programs.

The findings in this report are subject to at least five limitations. First, data provided by the Office of Vital Records are obtained via self-report and are subject to reporting bias. Second, residence, language and insurance data are obtained from audiologists and public health nurses, and the potential for reporting error exists. Third, other factors not part of this data set, such as comorbidities, might have affected the result. Fourth, some of the comparison groups have small numbers making it difficult to detect associations. Finally, because the outcome was studied as a dichotomous variable it was not possible to report relative delays associated with certain demographic factors.

Disparities in timely identification of hearing loss exist among infants who are DHH in Minnesota. Delayed identification might lead to delay in initiation of Early Intervention services which has been shown to result in poorer language outcomes in children identified as DHH. The information obtained from this study might help justify development of public health initiatives to target certain populations, including strengthening partnerships with local public health teams making home visits to MLBW and VLBW infants after hospital discharge. Another potential key partnership is with Special Supplemental Nutrition Programs for Women, Infants, and Children that have contact with low-income families, many of whom have public insurance. These teams are in a position to encourage or even facilitate scheduling of follow-up appointments for diagnostic hearing testing. Finally, creating information and resources for families in formats that are easily accessible but not dependent upon literacy or education levels (e.g., podcasts or online videos) is another public health initiative with the potential to improve outcomes.

**Summary**

**What is already known about this topic?**

The Joint Committee on Infant Hearing guidelines for Early Hearing Detection and Intervention recommends that all infants who have not passed newborn hearing screening should have diagnostic audiologic testing to identify hearing loss by age 3 months.

**What is added by this report?**

Significant delays in diagnosis of hearing loss among Minnesota infants identified as deaf or hard of hearing (DHH) were associated with low birth weight, lower maternal education, maternal age <25 years, maternal Hmong ethnicity, residence outside the metropolitan area, and public health insurance use.

**What are the implications for public health practice?**

Using this information to develop public health initiatives that target certain populations could improve timely identification, reduce the risk for language delay, and enhance outcomes in children who are DHH.
